# Heat shock proteins create a signature to predict the clinical outcome in breast cancer

**DOI:** 10.1038/s41598-019-43556-1

**Published:** 2019-05-17

**Authors:** Marta Klimczak, Przemyslaw Biecek, Alicja Zylicz, Maciej Zylicz

**Affiliations:** 1 International Institute of Molecular and Cell Biology, Warsaw, Poland; 2 Postgraduate School of Molecular Medicine, Medical University of Warsaw, Warsaw, Poland; 3 Faculty of Mathematics, Informatics, and Mechanics, University of Warsaw, Warsaw, Poland; 4 Faculty of Mathematics and Information Science, Warsaw University of Technology, Warsaw, Poland

**Keywords:** Breast cancer, Chaperones, Data processing

## Abstract

Utilizing The Cancer Genome Atlas (TCGA) and KM plotter databases we identified six heat shock proteins associated with survival of breast cancer patients. The survival curves of samples with high and low expression of heat shock genes were compared by log-rank test (Mantel-Haenszel). Interestingly, patients overexpressing two identified HSPs – HSPA2 and DNAJC20 exhibited longer survival, whereas overexpression of other four HSPs – HSP90AA1, CCT1, CCT2, CCT6A resulted in unfavorable prognosis for breast cancer patients. We explored correlations between expression level of HSPs and clinicopathological features including tumor grade, tumor size, number of lymph nodes involved and hormone receptor status. Additionally, we identified a novel signature with the potential to serve as a prognostic model for breast cancer. Using univariate Cox regression analysis followed by multivariate Cox regression analysis, we built a risk score formula comprising prognostic HSPs (HSPA2, DNAJC20, HSP90AA1, CCT1, CCT2) and tumor stage to identify high-risk and low-risk cases. Finally, we analyzed the association of six prognostic HSP expression with survival of patients suffering from other types of cancer than breast cancer. We revealed that depending on cancer type, each of the six analyzed HSPs can act both as a positive, as well as a negative regulator of cancer development. Our study demonstrates a novel HSP signature for the outcome prediction of breast cancer patients and provides a new insight into ambiguous role of these proteins in cancer development.

## Introduction

Despite the significant progress that has been made in recent years to improve the breast cancer diagnosis and treatment, it is still the most commonly diagnosed cancer among women and remains the second cancer-related death in women worldwide^1^. Breast cancer is a heterogenous group of tumors with distinct morphologies, clinical implications and response to therapy^2^. Patients are stratified into risk groups basing on the clinicopathological features (tumor size, lymph node stage, metastasis) combined with classical molecular features, such as the expression of estrogen receptor (ER), progesterone receptor (PR) and human epidermal growth factor receptor 2 (HER2)^3^. Currently, with the public availability of clinical and genomic data such as The Cancer Genome Atlas, lots of bioinformatics groups publish the multigene classifiers that could complement traditional diagnostic methods and develop more effective treatments. For example, association between physician characteristics and the use of 21-gene recurrence score genomic testing creates opportunities for breast cancer patients to receive optimal care^4^.

Heat shock proteins (HSPs) belong to a highly conserved family of proteins that act as molecular chaperones under stress conditions, including carcinogenesis^5,6^. They have been classified into the following families: HSPA (HSP70), HSPH (HSP110) HSPB (small heat shock proteins, sHSP), HSPC (HSP90), DNAJ (HSP40) and chaperonins^7,8^. HSPs interact with a broad range of unfolded, misfolded and semi-native proteins, assist in the acquisition of their active structures and prevent the formation of unwanted intermolecular interactions and protein aggregates^9–11^. In some cases, HSPs do not only prevent aggregation of proteins but also, using unfoldase activity, they are able to dissociate already existing protein aggregates^12–15^. Overexpression of HSPs has been observed in a wide range of human tumors, including breast, endometrial, ovarian, gastric, colon, lung and prostate cancers^5,16,17^. Most of previous studies reported correlation of high expression of HSPs with cancer aggressiveness and prognosis^6,17–19^. The overexpression of HSPs has been shown to be implicated in cancer cell proliferation, differentiation, invasion, metastasis and anti-apoptotic activity^16,18^. Recently, it was shown that heat shock proteins create a network which helps cells to survive stress conditions^20^. In cancer cells this network is remodeled to evade cell death, bypass senescence and refashion cell signaling to help highly malignant cancer cells to survive^11^. Moreover, it was shown recently that HSPs are involved in the evolution of cancer cells resulting in tumor heterogeneity. Such evolution could be accelerated by the chemotherapy and could lead to the acquisition of chemoresistance^11,21,22^. In contrast, some research has shown that reduced expression of HSPs is associated with poor prognosis in cancer patients^23,24^. Therefore, the biological mechanisms of HSPs and their role in cancer development still remain to be investigated.

In this study, we identified several heat shock genes which expression is crucial for breast cancer development. Interestingly, some of these HSPs work as negative regulators of cancer development and their expression is reduced in breast cancer cells, whereas other can support oncogenic activities and their expression in breast cancer cells is elevated. More importantly, identified HSPs that positively or negatively regulate breast cancer development, can play opposite role in other cancer types. The workflow of this study is presented in Supplementary Fig. S1.

## Results

### Identification of heat shock proteins associated with survival of breast cancer patients

**Figure 1:**
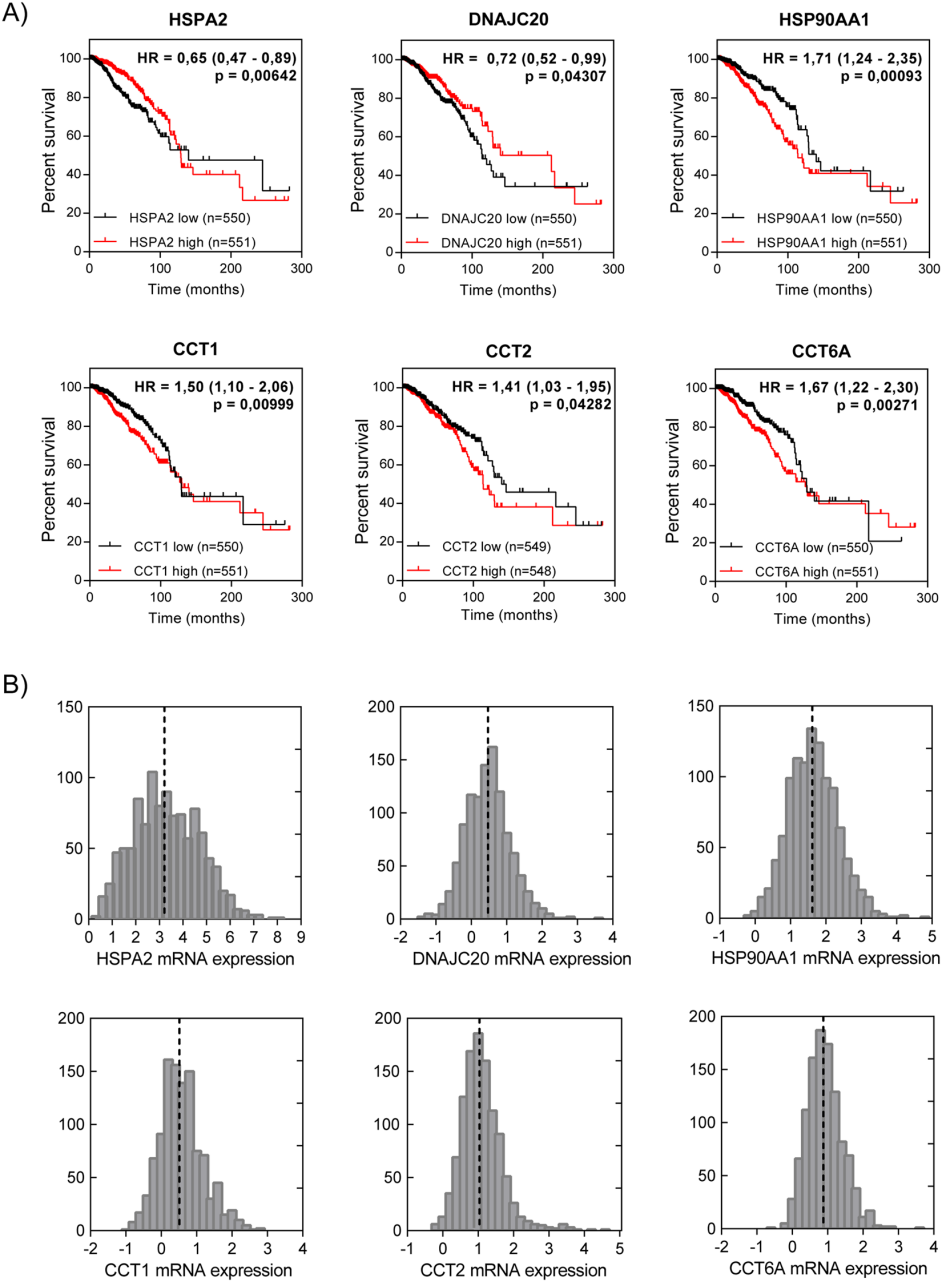
Expression of six identified HSPs predicts survival of breast cancer patients. (**A**) Kaplan-Meier survival curves of overall survival based on gene expression in cohort of TCGA BRCA patients. Hazard ratios (HR) with 95% confidence intervals and p-values (log-rank test, Mantel-Haenszel) were calculated. (**B**) Distribution of heat shock gene expression in TCGA BRCA dataset. The dotted lines indicate the median gene expression used as a cutoff. Normalized log2 mRNA data were obtained from XENA browser.

To identify the heat shock proteins (HSPs) associated with prognosis for breast cancer patients, we utilized TCGA and KM plotter datasets. We first performed the log-rank test (Mantel-Haenszel) to determine significant differences in patients’ survival depending on HSP expression. Expression of each of 96 HSPs was separated into low-expression and high-expression groups based on the median expression in each database used as a cutoff. We identified 13/96 HSPs (HSPA2, HSPA8, HSPA9, DNAJB5, DNAJC13, DNAJC20, DNAJC23, HSP90AA1, HSP90AB1, CCT1, CCT2, CCT4 and CCT6A) from the TCGA cohort and 22/96 HSPs (HSPA1A, HSPA1B, HSPA2, DNAJA1, DNAJC2, DNAJC5, DNAJC5G, DNAJC9, DNAJC16, DNAJC27, DNAJC20, HSPB1, HSPB5, HSP90AA1, CCT1, CCT2, CCT3, CCT5, CCT6A, CCT7, CCT8, HSP60) from KM plotter cohort significantly associated with overall survival (p ≤ 0,05). Six of them: HSPA2, DNAJC20, HSP90AA1, CCT1, CCT2 and CCT6A were statistically significant in both datasets (TCGA and KM plotter) and they were selected for further validation (Table 1). Comparison of the HSP expression in two independent datasets was performed to minimize the risk of false findings. With the exception of CCT6A, selected heat shock genes (HSPA2, DNAJC20, HSP90AA1, CCT1, CCT2) exhibited clinical significance also when subjected to univariate Cox regression model (Supplementary Fig. S2). Because expression of each of HSPs showed a nearly normal distribution, we divided patients into low-expression and high-expression groups by the median value (Fig. 1B). Interestingly, high expression of HSPA2 and DNAJC20 was significantly associated with better prognosis for breast cancer patients from TCGA cohort (p = 6,4e-03 and p = 4,3e-02, respectively), longer overall survival in KM plotter cohort (p = 4,5e-04 and p = 5,3e-03, respectively) and longer relapse-free survival in KM plotter cohort (p = 1,5e-07 and p = 5,3e-12, respectively) (Figs 1A, S3). In contrast, high expression of the other four HSPs (HSP90AA1, CCT1, CCT2, CCT6A) was significantly associated with reduced overall survival in TCGA cohort (p = 9,3e-04; p = 9,9e-03; p = 4,3e-02; p = 2,7e-03, respectively), reduced overall survival in KM plotter cohort (p = 9,4e-03; p = 1,6e-05; p = 1,4e-06; p = 6,7e-04, respectively) and reduced relapse-free survival in KM plotter cohort (p = 5,9e-15; p = 2e-09; p < 1e-16; p = 4,6e-15, respectively) (Figs 1A, S3). Collectively, these results suggest that high expression of HSPA2 and DNAJC20 is associated with low-risk breast cancer, whereas high expression of HSP90AA1, CCT1, CCT2 and CCT6A correlates with high-risk breast cancer.

**Table 1:** Association of 96 genes encoding heat shock proteins with overall survival of breast cancer patients from TCGA and KM plotter.

HSP family	Gene	OS (low vs high expression)		HSP family	Gene	OS (low vs high expression)	
		TCGA BRCA p-value	Kaplan_Meier plotter p-value			TCGA BRCA p-value	Kaplan_Meier plotter p-value
HSP70	HSPA1A	0,7898	0,0012**	HSP40	DNAJA1	0,1737	0,0104*
	HSPA1B	0,8009	0,0012**		DNAJA2	0,5296	0,062
	HSPA1L	0,2863	0,1784		DNAJA3	0,6468	0,7976
	**HSPA2**	0,00642**	0,00045***		DNAJA4	0,8641	0,19
	HSPA4	0,2382	0,1713		DNAJB1	0,1037	0,2173
	HSPA4L	0,9558	0,5283		DNAJB2	0,4024	0,0965
	HSPA5	0,642	0,3809		DNAJB3	N/A	N/A
	HSPA6	0,3907	0,478		DNAJB4	0,3863	0,6261
	HSPA7	0,6274	N/A		DNAJB5	0,03838*	0,6713
	HSPA8	0,04814*	0,0787		DNAJB6	0,7771	0,4362
	HSPA9	0,004824**	0,0562		DNAJB7	0,1804	0,0943
	HSPA12A	0,4018	0,7155		DNAJB8	N/A	0,2438
	HSPA12B	0,398	0,4443		DNAJB9	0,1394	0,2638
	HSPA13	0,8888	0,5764		DNAJB11	0,4576	0,958
	HSPA14	0,1735	0,1348		DNAJB12	0,6549	0,5622
HSP110	HSPH1	0,5553	0,054		DNAJB13	0,3657	0,0964
	HYOU1	0,445	0,5638		DNAJB14	0,4799	0,9681
HSPB	HSPB1	0,5245	0,003**		DNAJC1	0,7878	0,6448
	HSPB2	0,2765	0,5531		DNAJC2	0,5074	0,009**
	HSPB3	N/A	0,7777		DNAJC3	0,967	0,2065
	HSPB4	N/A	0,2363		DNAJC4	0,1096	0,0555
	HSPB5	0,05732	0,0211*		DNAJC5	0,1368	0,0294*
	HSPB6	0,5086	0,299		DNAJC5B	0,4402	0,8677
	HSPB7	0,2002	0,1558		DNAJC5G	N/A	0,0282*
	HSPB8	0,3012	0,7576		DNAJC6	0,07342	0,6954
	HSPB9	0,9359	0,1577		DNAJC7	0,719	0,304
	HSPB10	N/A	0,7791		DNAJC8	0,9024	0,6157
	HSPB11	0,4479	0,19		DNAJC9	0,6161	0,00000065****
HSPC (HSP90)	**HSP90AA1**	0,0009347***	0,0094**		DNAJC10	0,8865	0,4738
	HSP90AA3P	N/A	N/A		DNAJC11	0,4812	0,7795
	HSP90AB1	0,04601*	0,5055		DNAJC12	0,2195	0,0906
	HSP90B1	0,5217	0,3144		DNAJC13	0,01078*	0,5831
	HSP90L	0,1862	0,1497		DNAJC14	0,2178	0,2195
Chaperonins	BBS10	0,8031	0,2716		DNAJC15	0,5046	0,3538
	BBS12	0,4741	0,051		DNAJC16	0,2662	0,00000096****
	**CCT1**	0,009986**	0,000016****		DNAJC17	0,7579	0,0967
	**CCT2**	0,04282*	0,0000014****		DNAJC18	0,4269	0,6657
	CCT3	0,3363	0,0064**		DNAJC19	0,1643	0,0082
	CCT4	0,02085*	0,065		**DNAJC20**	0,04307*	0,0053**
	CCT5	0,09297	0,0064**		DNAJC21	0,445	0,6636
	**CCT6A**	0,002706**	0,00067***		DNAJC22	0,8126	0,7541
	CCT6B	0,6901	0,34		DNAJC23	0,001951**	0,056
	CCT7	0,1599	0,0119*		DNAJC24	0,7272	0,3404
	CCT8	0,223	0,00037***		DNAJC25	0,6785	0,8495
	HSPD1	0,5599	0,000004****		DNAJC26	0,2249	0,7234
	HSPE1	0,3572	0,0779		DNAJC27	0,9823	0,0038**
	MKKS	0,4983	0,4378		DNAJC28	0,3508	0,2808
					DNAJC29	0,9692	0,7073
					DNAJC30	0,2152	0,7974

Stratification into high-expression and low-expression groups was performed according to median expression of each HSP. P-value p ≤ 0,05 was considered as statistically significant. Six genes encoding HSPs marked in bold were identified as significantly associated with overall survival in both datasets and selected for further validation. *p ≤ 0,05; **p ≤ 0,01; ***p ≤ 0,001; ****p ≤ 0,0001.

### Breast cancer survival-associated HSPs are differentially expressed in normal and tumor tissue

**Figure 2:**
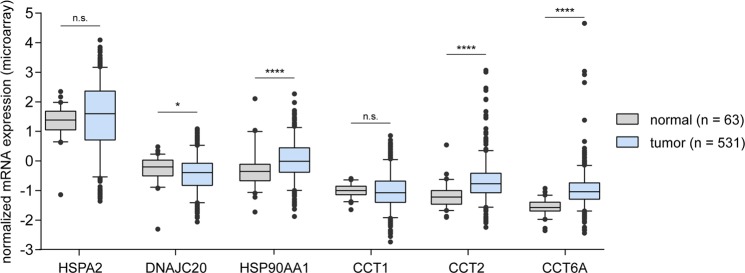
HSPs identified as associated with survival of breast cancer patients are differentially expressed in normal and tumor tissue. Box plots show the mRNA level of HSPs in primary breast cancer tissue (n = 531) and normal solid tissue (n = 63). Agilent array expression data for TCGA BRCA patients were obtained from XENA browser. Unpaired t test was used to calculate p-value. n.s. = not significant (p > 0,05); *p ≤ 0,05; **p ≤ 0,01; ***p ≤ 0,001; ****p ≤ 0,0001.

Differences in the expression of six HSPs between primary tumor tissue and normal solid tissue were assessed. The expression of DNAJC20 (better prognosis) was significantly lower in tumor tissue (log2 Fold Change (FC) = 0,7; p = 0,0251), whereas HSP90AA1, CCT2, CCT6A (unfavorable prognosis) were upregulated in tumors (HSP90AA1 log2 FC = 3,4; p < 0,0001; CCT2 log2 FC = 0,78; p < 0,0001; CCT6A log2 FC = 0,68; p < 0,0001, respectively). Analysis of HSPA2 and CCT1 expression did not reveal statistical difference between cancer and normal tissue (p > 0,05) (Fig. 2).

### The relationship between six HSP expression and clinicopathological features

To explore the effect of six prognostic HSPs on clinical features, we performed the analysis of each of HSP mRNA expression in subgroups stratified by clinicopathological features. We have observed that patients with high expression of HSPA2 (better prognosis) were associated with smaller tumors (p = 0,0162), ER-positive and PR-positive cancers (p < 0,0001 and p < 0,0001, respectively). Similarly, high expression of DNAJC20 (better prognosis) was observed in ER-positive and HER2-positive cancers (p < 0,0198 and p < 0,0001, respectively). In contrast, patients with increased expression of other four HSPs (poor prognosis) had higher clinical stage (HSP90AA1 p < 0,0001; CCT1 p = 0,0277; CCT2 p = 0,0005; CCT6A p = 0,0185), larger tumors (HSP90AA1 p < 0,0001; CCT1 p < 0,0001; CCT2 p = 0,0003; CCT6A p < 0,0001), more lymph nodes involved (HSP90AA1 p = 0,0139; CCT2 p = 0,003), ER-negative cancers (HSP90AA1 p = 0,0015; CCT1 p < 0,0001; CCT6A p < 0,0001), PR-negative cancers (HSP90AA1 p < 0,0001; CCT1 p < 0,0001; CCT6A p < 0,0001) and HER2-positive cancers (HSP90AA1 p < 0,0001; CCT1 p < 0,0001; CCT2 p = 0,0002; CCT6A p = 0,0075). Clinicopathological data of breast cancer patients are summarized in Table 2.

**Table 2:** Associations between six HSP expression and clinicopathological features of breast cancer patients.

Characteristic	No. of total cases	HSPA2 expression		DNAJC20 expression		HSP90AA1 expression		CCT1 expression		CCT2 expression		CCT6A expression	
		mean	p-value	mean	p-value	mean	p-value	mean	p-value	mean	p-value	mean	p-value
**Clinical stage**													
I	124	1,78	0,1453	0,42	0,583	1,55	**<** 0,0001****	0,51	0,0277*	1,06	0,0005***	0,89	0,0185*
II	358	1,45		0,37		1,82		0,66		1,17		1,05	
III & IV	130	1,61		0,35		1,95		0,69		1,33		1,03	
**Tumor**													
T1	210	1,78	0,0162*	0,40	0,4798	1,61	**<** 0,0001****	0,49	**<** 0,0001****	1,06	0,0003***	0,86	**<** 0,0001****
T2	469	1,39		0,36		1,87		0,71		1,25		1,09	
T3 & T4	109	1,43		0,41		1,86		0,68		1,26		1,01	
**Nodes**													
N0 & N1	649	1,52	0,6461	0,40	0,0828	1,77	0,0139*	0,64	0,3534	1,17	0,0030**	1,02	0,4735
N2 & N3	142	1,45		0,30		1,92		0,69		1,34		1,05	
**Metastasis**													
M0	771	1,49	0,1767	0,38	0,5855	1,80	0,6848	0,65	0,7587	1,21	0,4929	1,02	0,5778
M1	14	2,09		0,29		1,73		0,70		1,10		0,94	
**ER**													
positive	601	1,78	**<** 0,0001****	0,42	0,0198*	1,73	0,0015**	0,52	**<** 0,0001****	1,21	0,2401	0,90	**<** 0,0001****
negative	179	0,66		0,30		1,91		1,04		1,15		1,42	
**PR**													
positive	522	1,86	**<** 0,0001****	0,42	0,0641	1,71	**<** 0,0001****	0,50	**<** 0,0001****	1,20	0,942	0,88	**<** 0,0001****
negative	255	0,83		0,33		1,92		0,92		1,21		1,29	
**HER2**													
positive	114	1,45	0,6285	0,58	0,0001****	2,11	**<** 0,0001****	0,87	**<** 0,0001****	1,37	0,0002***	1,15	0,0075**
negative	652	1,54		0,35		1,73		0,61		1,16		1,00	

Expression of individual HSP was compared between cohorts stratified by clinicopathological features using unpaired t test (for two groups) or one-way ANOVA (for three groups). n.s. = not significant (P > 0,05); *P ≤ 0,05; **P ≤ 0,01; ***P ≤ 0,001; ****p ≤ 0,0001. Data for TCGA BRCA patients were obtained from XENA browser.

**Figure 3:**
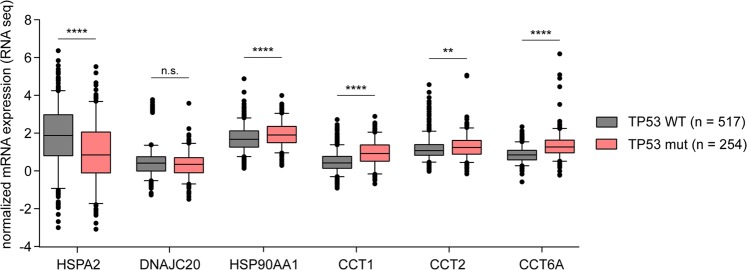
Association between TP53 status and mRNA expression level of six HSPs. HSP mRNA expression levels were compared between samples with TP53 wild type (TP53 WT) and mutant (TP53 mut) forms. RNAseq data for TCGA BRCA patients were obtained from XENA browser. Unpaired t test was used to calculate p-value. n.s. = not significant (p > 0,05); *p ≤ 0,05; **p ≤ 0,01; ***p ≤ 0,001; ****p ≤ 0,0001.

It is well established that tumor suppressor encoded by TP53 gene is at the crossroads of a network of signaling pathways that prevents cancer development^11^. Moreover, mutated TP53 lose the oncosuppressive role and acquire new oncogenic functions. In line with this, we found significantly increased expression of HSPA2 (better prognosis) in TP53 WT cancers (log2 FC = 0,99; p < 0,0001), whereas high expression of HSP90AA1, CCT1, CCT2 and CCT6A (poor prognosis) coincided with TP53 mutations (HSP90AA1 log2 FC = 0,18; p < 0,0001; CCT1 log2 FC = 0,99; p < 0,0001; CCT2 log2 FC = 0,15; p = 0,0047; CCT6A log2 FC = 0,65; p < 0,0001, respectively) (Fig. 3).

### Development of prognostic signature based on the expression of HSPs to predict the survival of breast cancer patients

**Figure 4:**
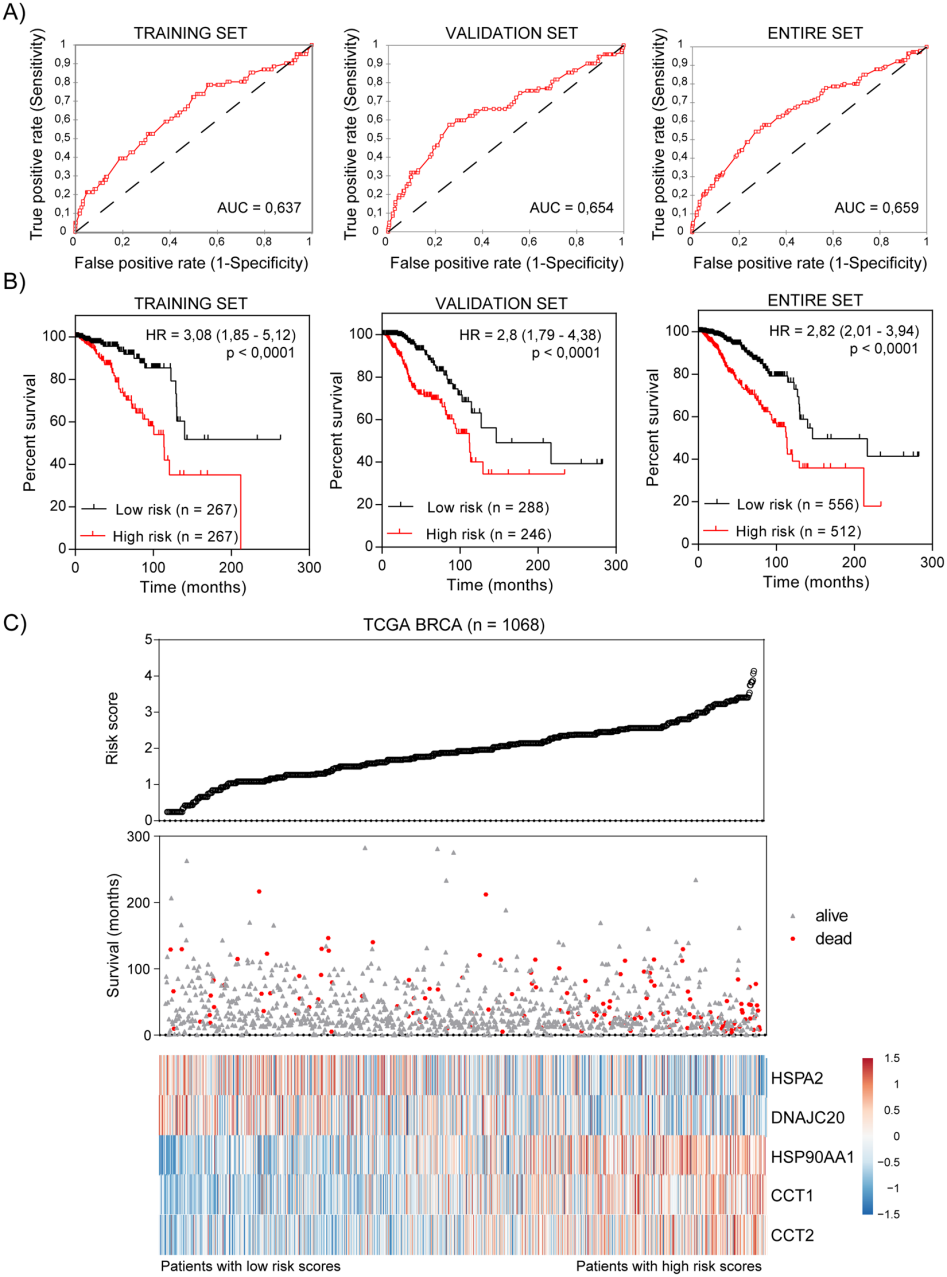
Signature for survival prediction of breast cancer patients. (**A**) Diagnostic value of five candidate HSPs and cancer stage in the training (n = 534), validation (n = 534) and entire TCGA BRCA dataset (n = 1068). The areas under curve (AUC) were calculated for ROC curves, and sensitivity and specificity were calculated to assess the score performance. (**B**) Kaplan-Meier survival curves for five-HSP and stage signature in the training (n = 534), validation (n = 534) and entire TCGA BRCA dataset (n = 1068). Patients were stratified into high-risk and low-risk groups based on median of risk score. Hazard ratios (HR) with 95% confidence intervals and log-rank test p-values were calculated. (**C**) The signature-based risk score distribution, patients’ survival status and heatmap of five HSP expression profiles. Blue and red values represent down- and upregulation, respectively. mRNA expression Z-scores for TCGA BRCA patients were obtained from cBioPortal.

To build a prediction model, evaluated previously HSPs as well as clinical candidate predictors (stage, ER status, PR status and HER2 status) were subjected to univariate Cox regression model. In total, five HSPs and stage of cancer were significantly correlated with the overall survival of breast cancer patients (p < 0,05; Table 3). Two of HSPs (HSPA2, DNAJC20) had negative coefficients, suggesting that their higher expression was observed in patients with longer survival. The positive coefficients for the remaining three significant HSPs (HSP90AA1, CCT1, CCT2) represented that the higher expression level was observed in patients with poor survival. As expected, cancer stage exhibited a positive coefficient indicating a worse prognosis. One of HSPs and receptors were not prognostically relevant for overall survival (in univariate analysis p > 0,05) and were omitted from further prognosis evaluation. The 1068 breast invasive carcinoma (BRCA) patients from TCGA dataset were randomly divided into a training set (n = 534) and a validation set (n = 534). Basing on the expression level of five prognostic HSPs, cancer stage and multivariate Cox regression coefficients for training set, we built a risk score formula for BRCA patients’ survival prediction. Expression data were converted to a binary format (low expression = 0, high expression = 1) and cancer stage data (AJCC_PATHOLOGIC_TUMOR_STAGE) were converted as follows: Stage I, IA, IB = 1; Stage II, IIA, IIB = 2; Stage III, IIIA, IIIB, IIIC = 3; Stage IV = 4. Patients with Stage X and NA were excluded from the analysis. Risk score was constructed with the formula: Risk score = (−0,4181 × HSPA2 0/1) + (−0,1813 × DNAJC20 0/1) + (0,6861 × HSP90AA1 0/1) + (0,0824 × CCT1 0/1) + (0,11 × CCT2 0/1) + (0,8427 × Stage 1/2/3/4). We next validated our signature in the validation set to confirm our findings. By calculating the risk score for each patient in the validation set based on the same risk score formula, we divided BRCA patients into a low-risk group (n = 290) and high-risk group (n = 244) using the same threshold. The risk score showed a great survival prediction in breast cancer with area under curve (AUC) equal to 0,6237 in the training set, AUC equal to 0,654 in the validation set, AUC equal to 0,659 in entire BRCA cohort and AUC equal to 0,572 in independent METABRIC dataset (Figs 4A, S4). The Kaplan-Meier curve suggested that patients in the high-risk group suffered worse prognosis than patients in the low-risk group (median survival 100,6 months vs 212,1 months, p < 0,0001 in the training set; median survival 112,3 vs 216,6 months, p < 0,0001 in the validation set; median survival 112,3 vs 212,1, p < 0,0001 in the entire TCGA dataset) (Fig. 4B). The distribution of the risk score, patients’ survival status and expression profiles of prognostic HSPs were ranked according to the risk score value (Fig. 4C). Patients with a high risk score had greater mortality than patients with low risk score (Fig. 4C, middle panel). In addition, patients with a high-risk score had higher expression of HSP90AA1, CCT1 and CCT2, whereas the expression of the remaining two HSPs (HSPA2 and DNAJC20) was downregulated (Fig. 4C, heatmap). These findings suggested that risk score calculated basing on five HSP expression and stage of cancer has a competitive performance for the survival prediction of BRCA patients (Supplementary Fig. S5). Importantly, nearly identical results of survival prediction were obtained when Stage variable was binarized (Stage I 0/1, Stage II 0/1, Stage III 0/1) and coefficients in a Cox regression model were calculated for each Stage (Supplementary Fig. S6).

**Table 3:** Univariate and multivariate Cox proportional hazards analysis of overall survival for TCGA BRCA patients.

Univariate analysis					
Variable	Coefficient	p-value	HR	95% CI	
				Lower	Upper
**HSPA2**	−0,148	**0,012**	0,863	0,769	0,969
**DNAJC20**	−0,296	**0,027**	0,744	0,572	0,967
**HSP90AA1**	0,369	**0,001**	1,447	1,157	1,809
**CCT1**	0,506	**0,000**	1,659	1,283	2,145
**CCT2**	0,290	**0,020**	1,337	1,046	1,708
CCT6A	0,135	0,384	1,145	0,845	1,553
**STAGE**	0,736	**<** **0,0001**	2,087	1,668	2,610
ER	−0,325	0,081	0,722	0,501	1,041
PR	−0,299	0,082	0,741	0,529	1,039
HER2	0,472	0,058	1,603	0,984	2,611
**Multivariate analysis**					
Variable	Coefficient	p-value	HR	95% CI	
				Lower	Upper
**Training set (n** = **534)**					
HSPA2	−0,418	0,114	0,658	0,392	1,106
DNAJC20	−0,181	0,500	0,834	0,493	1,413
HSP90AA1	0,686	**0,038**	1,986	1,040	3,793
CCT1	0,082	0,772	1,086	0,622	1,897
CCT2	0,110	0,734	1,116	0,593	2,103
STAGE	0,843	**<** **0,0001**	2,323	1,566	3,446
**Validation set (n = 534)**					
HSPA2	−0,517	**0,039**	0,597	0,366	0,974
DNAJC20	−0,070	0,774	0,933	0,581	1,499
HSP90AA1	0,357	0,174	1,429	0,855	2,388
CCT1	0,502	**0,046**	1,653	1,009	2,708
CCT2	−0,165	0,519	0,848	0,515	1,398
STAGE	0,802	**<** **0,0001**	2,230	1,635	3,041
**Entire TCGA set (n = 1068)**					
HSPA2	−0,460	0,010	0,631	0,445	0,896
DNAJC20	−0,125	0,480	0,882	0,623	1,249
HSP90AA1	0,477	**0,019**	1,612	1,082	2,400
CCT1	0,320	0,082	1,378	0,960	1,978
CCT2	−0,022	0,912	0,979	0,666	1,438
STAGE	0,793	**<** **0,0001**	2,211	1,742	2,806

HR, hazard ratio; CI, confidence interval.

### Functional characteristics of HSP prognostic signature

**Figure 5:**
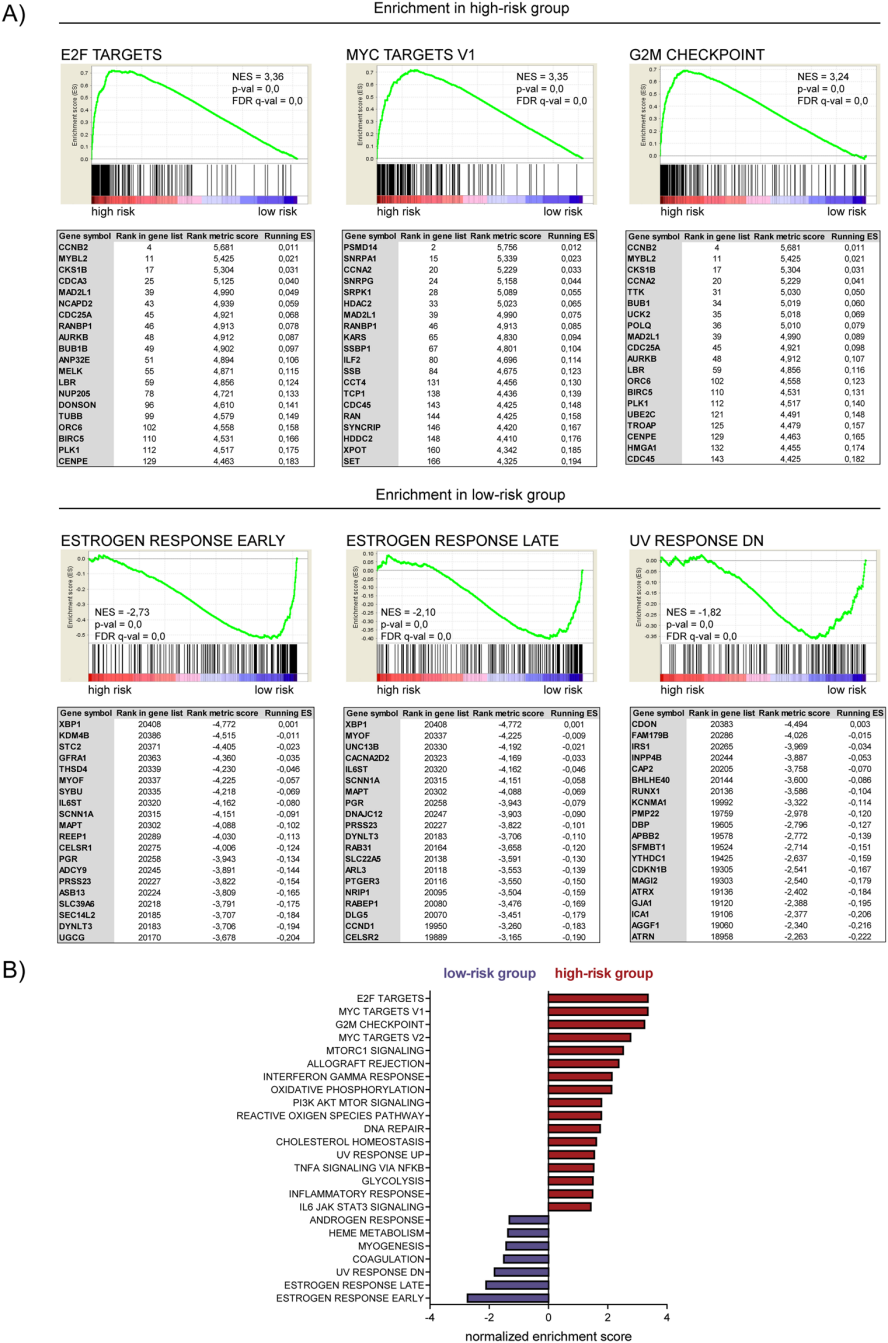
GSEA results for high-risk and low-risk groups. (**A**) GSEA plots of three most significantly enriched datasets in high-risk (top panel) or low-risk (bottom panel) groups are shown. The tables enumerate the genes in the pathway which were the most significantly enriched in high-risk versus low-risk group (top panel) or low-risk versus high-risk group (bottom panel). NES (normalized enrichment score), p-val (nominal p-value), FDR q-val (false discovery rate). (**B**) Normalized enrichment scores for GSEA analysis of MSigDB hallmark gene sets enriched in high-risk (RED) or low-risk (VIOLET) groups. Gene sets with p ≤ 0,05 and FDR ≤ 0,25 were shown.

To explore the functional implications of five-HSP signature, we performed gene set enrichment analysis (GSEA). The top 3 enriched datasets from GSEA analysis were shown in Fig. 5A. We found that the most upregulated genes in high-risk group clustered most significantly in cell-cycle associated processes including *E2F targets* (NES = 3,36)*, MYC targets* (NES = 3,35)*, G2M checkpoint* (NES = 3,24) (Fig. 5A, top panel). In contrast, the most enriched processes in low-risk group included *estrogen response early* (NES = −2,73)*, estrogen response late* (NES = −2,10)*, UV response DN* (NES = −1,82) (Fig. 5A, bottom panel). All processes enriched in high-risk or low-risk group were mentioned in Fig. 5B.

### HSPs associated with breast cancer survival play dual roles in other cancer types

**Figure 6:**
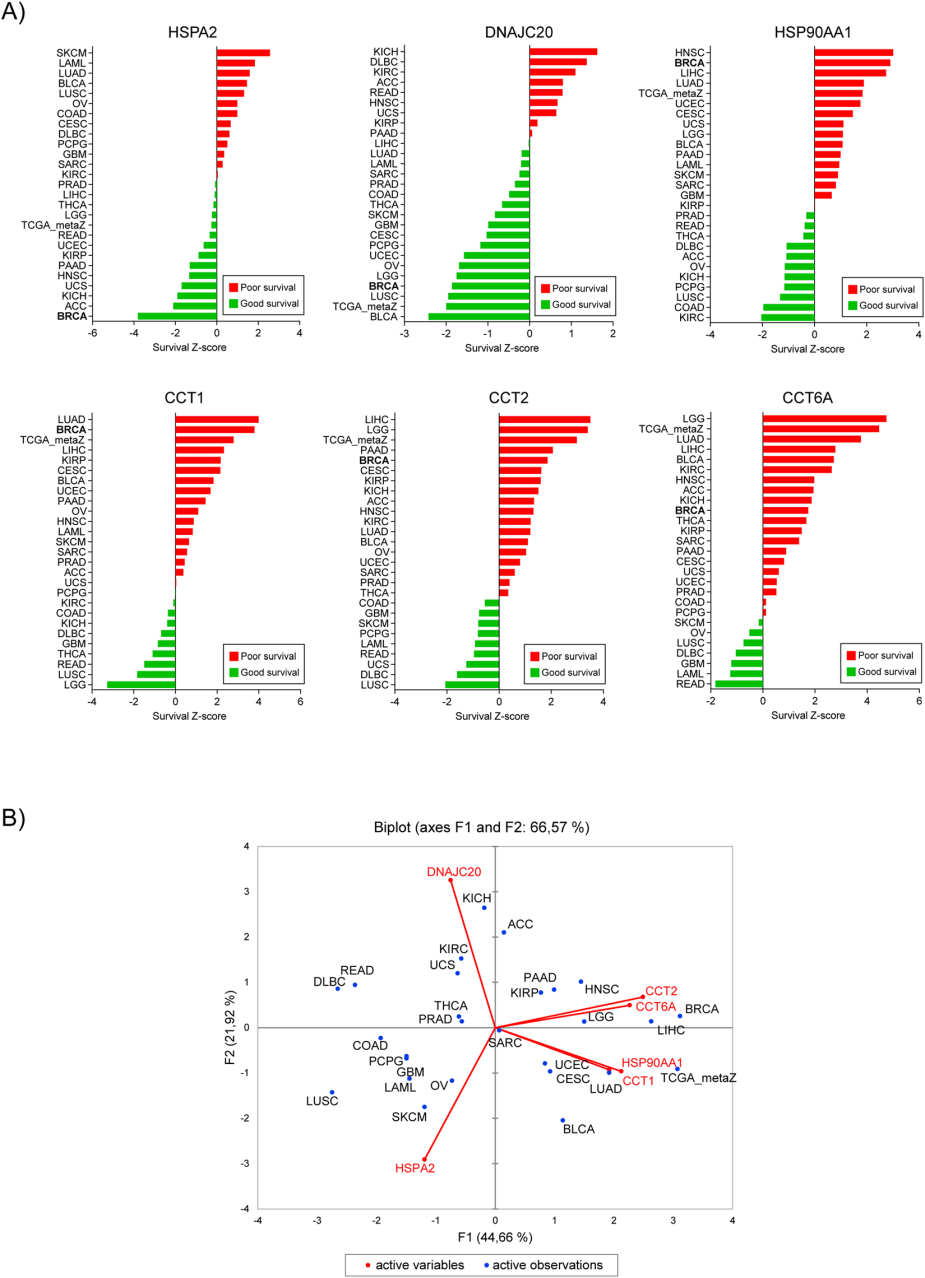
HSPs play distinct role in different cancer types. (**A**) Survival Z-scores in different cancer types associated with expression of HSP mRNA. Positive and negative Z-scores reflect association between high expression of given HSP and poor (red) or good (green) prognosis for cancer patients, respectively. The data were obtained from PRECOG tool (http://precog.stanford.edu). ACC - Adrenocortical carcinoma, BLCA - Bladder Urothelial Carcinoma, BRCA - Breast invasive carcinoma, CESC - Cervical squamous cell carcinoma and endocervical adenocarcinoma, COAD - Colon adenocarcinoma, DLBC - Lymphoid Neoplasm Diffuse Large B-cell Lymphoma, GBM - Glioblastoma multiforme, HNSC - Head and Neck squamous cell carcinoma, KICH – Kidney Chromophobe, KIRC - Kidney renal clear cell carcinoma, KIRP - Kidney renal papillary cell carcinoma, LAML - Acute Myeloid Leukemia, LGG - Brain Lower Grade Glioma, LIHC - Liver hepatocellular carcinoma, LUAD - Lung Adenocarcinoma, LUSC - Lung squamous cell carcinoma, OV - Ovarian serous cystadenocarcinoma, PAAD - Pancreatic adenocarcinoma, PCPG - Pheochromocytoma and Paraganglioma, PRAD - Prostate adenocarcinoma, READ - Rectum adenocarcinoma, SARC - Sarcoma, SKCM - Skin Cutaneous Melanoma, TCGA_metaZ – global meta-Z-score in all TCGA cancer types, THCA - Thyroid carcinoma, UCEC - Uterine Corpus Endometrial Carcinoma, UCS - Uterine Carcinosarcoma. (**B**) Biplot showing the principal component analysis (PCA) of relationship between six HSP expression (mRNA expression Z-scores for HSPA2, DNAJC20, HSP90AA1, CCT1, CCT2, and CCT6A; marked with arrows) and overall survival in different types of cancer (marked as points).

As heat shock proteins are mostly reported to play pro-oncogenic role in cancer development, we utilized PRECOG (PREdiction of Clinical Outcomes from Genomic Profiles) tool to investigate the association between six HSP expression and overall survival in various solid and liquid cancers. For HSPA2 we observed correlation with both good and bad prognosis depending on cancer types. The poor survival (survival Z-score > 0) associated with HSPA2 overexpression was observed for Skin Cutaneous Melanoma (SKCM), Acute Myeloid Leukemia (LAML), Lung adenocarcinoma (LUAD), Bladder Urothelial Carcinoma (BLCA), Lung squamous cell carcinoma (LUSC), Ovarian serous cystadenocarcinoma (OV), Colon adenocarcinoma (COAD), Cervical squamous cell carcinoma and endocervical adenocarcinoma (CESC), Lymphoid Neoplasm Diffuse Large B-cell Lymphoma (DLBC), Pheochromocytoma and Paraganglioma (PCPG), Glioblastoma multiforme (GBM) and Sarcoma (SARC), whereas the favorable prognosis (survival Z-score < 0) was observed for Breast invasive carcinoma (BRCA), Adrenocortical carcinoma (ACC), Kidney Chromophobe (KICH), Uterine Carcinosarcoma (UCS), Head and Neck squamous cell carcinoma (HNSC), Pancreatic adenocarcinoma (PAAD), Kidney renal papillary cell carcinoma (KIRP), Uterine Corpus Endometrial Carcinoma (UCEC), Rectum adenocarcinoma (READ), Brain Lower Grade Glioma (LGG), Thyroid carcinoma (THCA), Liver hepatocellular carcinoma (LIHC) and Prostate adenocarcinoma (PRAD). High expression of DNAJC20 correlated with good prognosis (survival Z-score < 0) for most of cancer types excluding KICH, DLBC, KIRC, ACC, READ, HNSC, UCS, KIRP, PAAD. Conversely, the high expression of other four HSPs (HSP90AA1, CCT1, CCT2, CCT6A) was associated with poor prognosis (survival Z-score > 0) for most types of cancer. HSP90AA1 correlated with good prognosis only in PRAD, READ, THCA, DLBC, AAC, OV, KICH, PCPG, LUCS, COAD and KIRC. CCT1 also played pro-oncogenic role in most cancer types excepting KIRC, COAD, KICH, DLBC, GBM, THCA, READ, LUSC and LGG. Similar results were observed when we correlated expression of CCT2 with clinical outcome. In most cancer types, CCT2 correlated with poor prognosis, but there were some like COAD, GBM, SKCM, PCPG, LAML, READ, UCS, DLBC and LUCS which had increased survival rate when CCT2 was overexpressed. Correlation between high expression of CCT6A and good survival was observed just for 7/26 cancer types including SKCM, OV, LUSC, DLBC, GBM, LAML and READ (Fig. 6A).

In summary, we have shown that overexpression of HSPA2 and DNAJC20 in most cancer types correlates with favorable prognosis suggesting tumor suppressor activity of these gene products whereas high expression of HSP90AA1, CCT1, CCT2 and CCT6A correlates mainly with poor prognosis suggesting oncogenic activity of these gene products (Fig. 6B).

## Discussion

Detailed transcriptomic analysis of several different types of tumors and their normal counterparts (TCGA database) shows that in cancer cells, genes conserved with unicellular organisms were strongly up-regulated, whereas genes of metazoan origin were primarily inactivated. Moreover, the coordinated expression of strongly interacting multicellularity and unicellularity processes was lost in tumors^25^. It has been shown previously that HSPs belong to the highly conserved network which helps to survive both unicellular and multicellular organisms^11^. Several mechanisms are involved in the cytoprotective effect of HSPs: 1 – as molecular chaperones, HSPs catalyze the proper folding of new proteins and prevent formation of potential aggregates in existing structures^26^; 2 – expression of HSPs correlates with increased resistance to apoptosis revealing their prosurvival mechanism^27,28^; 3 – HSPs favour the proteasomal degradation of certain proteins under stress conditions^29,30^. In the case of cancer cells, these HSP networks are extensively remodeled in such a way that they become advantageous to the proliferating cells, i.e. misregulation of the stress signaling cascades, receptor blocking or hiperactivation, effective apoptosis and senescence evasion^11,20^. The crucial role of HSPs in cell transformation and tumor evolution leads to the consideration that HSPs are important therapeutic targets for cancer treatment^11,16,17,31^.

Herein, using The Cancer Genome Atlas and KM plotter database, we showed strong association between expression of at least six HSP-encoding genes (namely: HSPA2, DNAJC20, HSP90AA1, CCT1, CCT2 and CCT6A) and survival of breast cancer patients. Interestingly, among these HSPs, overexpression of HSPA2 and DNAJC20 was associated with better prognosis (tumor suppressor-like activity), whereas high expression of other heat shock genes (HSP90AA1, CCT1, CCT2 and CCT6A) correlated with poor survival (oncogene-like activity). These results are in line with the recent comprehensive study of Zoppino *et al*. demonstrating expression of among others: HSPA2, DNAJC20, HSP90AA1, CCT1, CCT2 with significance on survival^32^.

The HSPA2 belongs to the HSPA/HSP70 family of proteins possessing ATPase activity required for their molecular chaperone activity. Specificity of these activities towards different substrates is driven by DNAJ specificity factors^33,34^. In our study, the emergence of the tumor suppressive functions of HSPA2 was supported by the decreasing HSPA2 expression in larger and more advanced tumors. Additionally, higher expression of HSPA2 was observed in ER- and PR-positive breast cancers which are linked to better clinical outcome. Significantly elevated expression of HSPA2 was observed in tumors with no mutation in tumor suppressor TP53 preventing the tumor development and metastasis. However, according to previously reported studies, overexpression of HSPA2 was also associated with worse clinical outcome. HSPA2 (HSP70-2) is expressed at high levels in testis where it plays an essential role in spermatogenesis and has been described as an important biomarker in many cancer types^35^. High expression of HSPA2 have been associated with shorter overall survival in stage I-II of non-small cell lung carcinoma patients^36^. HSPA2 was also overexpressed in esophageal squamous cell carcinoma and was significantly associated with primary tumor, TNM stage, lymph node metastases and recurrence resulting in shorter DFS and OS^37^. Similarly, increased HSPA2 in pancreatic ductal adenocarcinoma and hepatocellular carcinoma was associated with more aggressive clinical features and shorter overall survival^38,39^. Contrary to our findings, a recent study indicated that HSPA2 might also play an essential role in breast cancer development and progression by promoting cell growth and cellular motility both in culture as well as *in vivo* in xenotransplanted mice^40^. On the other hand, overexpression of HSPA2 was found to correlate with longer overall survival in breast cancer patients basing on the TCGA data, the Netherlands Cancer Institute (NKI) data and data from several other breast cancer gene expression datasets at the Oncomine (http://www.oncomine.org/)^24,32^. These observations could suggest the dual role of HSPA2, both tumor suppressive and prosurvival, in different cancer types as well as the possibility of some limitations resulting from dish-based culture which does not fully develop the tumor microenvironment conditions.

Another survival-associated HSP protein identified in our study is DNAJC20. This protein belongs to the DNAJ family which functions as substrate specificity factors for HSPA/HSP70 family. DNAJC20 acts as a co-chaperone in iron-sulfur cluster biosynthesis in mitochondria^41^. To the best of our knowledge, no clear evidence for the involvement of DNAJC20 in cancer development has been presented before. Basing on the TCGA data from microarray analysis, we observed decreased expression of this heat shock gene in tumors when compared to normal tissues. In addition, low expression of DNAJC20 correlated with poor survival of breast cancer patients. Decreased expression of DNAJC20 was associated with ER-negative and HER2-negative tumors suggesting correlation of low expression of DNAJC20 with more aggressive basal breast cancer subtype^42^.

In this study, we identified HSP90AA1 gene encoding HSP90 alpha protein, the inducible isoform of HSP90, among four most significant factors of poor prognosis in breast cancer. Indeed, in previous reports it has been described that HSP90 (both HSP90α and HSP90β) increases the risk of recurrence and distant metastases in triple negative and ER+/HER2- breast cancer subtypes, as well as strongly associates with the risk of death from breast cancer^43^. Another studies indicated overexpression of HSP90α in human breast cancer cells associated with increased cell proliferation^44^. HSP90 is also involved in many cancer-associated processes like cellular transformation^45^, DNA double-strand break repair^46,47^, apoptosis^48^, invasion^49^, genetic variation^50,51^. Due to the complex involvement in oncogenic signaling, HSP90α has attracted much attention as a potential therapeutic target. Not surprisingly, we also observed strong correlation between HSP90AA1 expression and poor survival of breast cancer patients based on data from two databases. Consistent with previous observations, HSP90AA1 was overexpressed in tumors when compared to normal tissue. Additionally, overexpression of HSP90AA1 was observed in tumors containing mutation in TP53, one of the most frequent genetic alteration in cancer that is often associated with accelerated tumor progression. Oncogenic properties of HSP90α correlated with aggressive clinicopathological features including high clinical stage, large tumors (T3 & T4) and lymph node involvement.

Intriguingly, next three genes identified in our studies, which high expression correlates with poor prognosis of breast cancer patients, are the subunits of molecular chaperonin complex CCT/TRiC (CCT for chaperonin containing TCP1, also called TCP-1 ring complex). CCT/TRiC complex consists of two rings stacked back-to-back, each ring is composed of eight distinct subunits (CCT1-CCT8)^52,53^. CCT has been shown to mediate folding of approximately 10% of the eukaryotic proteome including a number of cancer-linked proteins like p53^54^, tumor suppressor Von Hippel-Lindau^55^, signal transducer and activator of transcription 3 (STAT3)^56^, cyclin E^57^, p21^Ras^ and cyclin B^58^. High expression of CCT2 occurred in liver, prostate and breast cancer and correlated with cancer severity and unfavorable prognosis^59,60^. Another study reported that CCT1 and CCT2 were amplified in breast cancer and necessary for cell survival and growth^61^. CCT subunits have been also implicated in the development of hepatocellular carcinoma^62,63^, gastric^64^, esophageal^65^ and colon cancer^66^. According to our studies, three subunits of CCT complex – CCT1, CCT2 and CCT6A strongly correlated with survival of breast cancer patients. Tumor samples showed a significantly higher expression of these subunits than normal controls. Moreover, high expression of identified CCT subunits was associated with aggressive clinical features including the high stage and grade of cancer. Increased expression of CCT subunits negatively correlated with the status of estrogen (ER) and progesterone (PR) receptors indicating more aggressive cancers. The involvement of particular subunits of CCT complex in tumorigenesis observed in our studies and previously reported in different types of cancer, raise the question of whether CCT subunits exert tumorigenic effects acting as independent monomers or components of CCT complex. In fact, there are several studies showing that some individual subunits of CCT chaperonin, when monomeric, can have an oligomer-independent functions^67–71^. On the other hand, subunits of CCT complex are thought to recognize different motifs in substrates^52^. Therefore, even if they are bound in a CCT complex, they may recruit specific clients involved in the regulation of oncogenesis. To date, it still remains unclear whether the pro-oncogenic role of CCT complex results from the properties of its individual components or the full complex.

In this study, we revealed ambiguous role of HSPs in various types of cancer. We identified that depending on cancer type, each of the analyzed HSPs can act both as a positive as well as a negative regulator of carcinogenesis. These findings explain the semi-contradictory reports in the literature. A prosurvival role of HSPs have been reported several times^11^, but the positive correlation between expression of HSPs and better prognosis provides very new insight into the role of molecular chaperones in tumorigenesis. Besides our studies, there are only a few reports of tumor suppressive functions of HSPs^23,24,32^.

Finally, by using univariate Cox regression analysis followed by multivariate Cox regression analysis, we identified HSP expression signature combined with tumor stage that was associated with survival of breast cancer patients. Then, by calculating a risk score and performing ROC curve analysis, we found that this signature demonstrated significant prognostic performance in training, validation and entire TCGA dataset. Utilizing our risk score and GSEA analysis, we observed that high-risk patient cohort was enriched in cell cycle regulators whereas low-risk group overexpressed genes involved in estrogen response suggesting less aggressive luminal subtypes of breast cancer.

In conclusion, our study investigates the involvement of heat shock proteins in breast cancer development and contributes to the comprehension of the complex role of these proteins in other cancer type. Our unpublished results demonstrate the influence of six identified HSPs on proliferation, viability and response to chemotherapy in various breast cancer cell lines. Further functional investigations are needed to validate our studies and elucidate the molecular mechanisms underlying the role of these identified HSPs in tumorigenesis. Nevertheless, our study might be helpful to predict the survival of breast cancer patients and serves as an inspiration for seeking of potential new targets in cancer treatment.

## Methods

### Patient cohorts

All 1247 patients of breast invasive carcinoma (BRCA) were retrieved from The Cancer Genome Atlas (TCGA) and downloaded from the UCSC Xena browser (http://xena.ucsc.edu) or The cBioPortal for Cancer Genomics (http://cbioportal.org)^72,73^. Normal and metastatic samples have been excluded from analysis. Overall, 1101 TCGA samples of primary tumor have been included in our study with the corresponding clinical information and gene expression data. To validate the survival results obtained from TCGA dataset, we used an online database KM plotter (http://kmplot.com/analysis/) which contains data of 5143 breast cancer patients^74^. In this database, gene expression data, relapse free survival and overall survival information have been downloaded from Gene Expression Omnibus (GEO, Affymetrix microarrays only), European Genome-phenome Archive (EGA) and TCGA.

### Survival analysis

Breast cancer patients from TCGA dataset were divided into high-expression and low-expression groups by the median values of mRNA expression. Significant differences in survival were assessed by log-rank (Mantel-Haenszel) test using GraphPad Prism 6. P-value less than 0,05 was considered as statistically significant. Survival curves for BRCA patients from KM plotter were generated on the webpage. Hazard ratios (HR) and p-values (from the log-rank test) were calculated online. Then, HSPs were fitted in a univariate and multivariate Cox proportional hazards regression analysis using R software. Risk scores were estimated by involving selected HSPs and cancer stage, which where weighted by their estimated regression coefficients in the multivariate Cox regression model. Patients were divided into high-risk and low-risk groups using the median risk score as a cutoff value. Differences in patient survival between these two groups were estimated by the Kaplan-Meier survival analysis and log-rank (Mantel-Haenszel) test. The receiver operating characteristic (ROC) curve for the risk score and survival status (0 – deceased, 1 – living) was performed in XLSTAT statistical software to assess the predictive accuracy of prognostic model.

### TCGA database analysis

Box plots of HSP expression in normal/cancer tissue of TCGA BRCA patients were generated using pan-cancer normalized Agilent array expression from XENA browser. Statistics was calculated using unpaired t test in GraphPad Prism 6. For box plots comparing TP53 status in low-expression and high-expression groups, we used gene expression RNAseq data (normalized_log2[norm_count + 1]) from XENA browser. Statistics was calculated using unpaired t test in GraphPad Prism 6. The association of HSP expression and clinicopathological features presented in Table 2 have been analyzed using gene expression RNAseq data (normalized_log2[norm_count + 1]) and clinical traits from XENA browser. P-value has been calculated using unpaired t test or one-way ANOVA in GraphPad Prism 6. Heatmaps were generated using ClustVis web tool (http://biit.cs.ut.ee/clustvis/) and gene expression RNAseq data (mRNA median Z-score) from cBioPortal^72,73,75^.

### PRECOG analysis

Survival Z-scores for individual genes and cancer types from TCGA were obtained from the PREdiction of Clinical Outcomes from Genomic Profiles (PRECOG) portal (http://precog.stanford.edu)^76^. PRECOG encompasses 165 cancer expression datasets, including overall survival data for ~26,000 patients diagnosed with 39 distinct malignancies. Survival Z-scores have been calculated for the whole TCGA dataset and for 26 individual cancer types from TCGA.

### Functional enrichment analysis

Gene Set Enrichment Analysis (GSEA) software version 3.0 from the Broad Institute was used to identify significantly enriched gene sets^77,78^. BRCA patients from TCGA cohort were dichotomized into low-risk and high-risk groups based on the median of risk score. Our input file contained expression data for 20437 genes and 1100 patients. We used 1000 gene set permutations for the analysis and pathways with nominal p-value p ≤ 0,05 and FDR ≤ 0,25 were considered significant. We used 50 pathways in the hallmark gene sets (H) collection from MSigDB.

## Supplementary information

Supplementary Information
