# Supplementary material for: Heat shock proteins create a signature to predict the clinical outcome in breast cancer
Source: Sci Rep. 2019 May 17;9:7507. doi: 10.1038/s41598-019-43556-1 (PMC6525249; doi:10.1038/s41598-019-43556-1)
Supplement: Supplementary file 1 — Supplementary Information [file 41598_2019_43556_MOESM1_ESM.pdf]

# Supplementary Information

# Heat shock proteins create a signature to predict the clinical outcome in breast cancer

**Marta Klimczak[1,2]*, Przemysław Biecek[3,4], Alicja Zylicz[1], Maciej Zylicz[1]***

[1]International Institute of Molecular and Cell Biology, Warsaw, Poland
[2]Postgraduate School of Molecular Medicine, Medical University of Warsaw, Warsaw, Poland
[3]Faculty of Mathematics, Informatics, and Mechanics, University of Warsaw, Warsaw, Poland
[4]Faculty of Mathematics and Information Science, Warsaw University of Technology, Warsaw, Poland

*Correspondence and requests for materials should be addressed to M.K. and M.Z. (email: mklimczak@iimcb.gov.pl; mzylicz@iimcb.gov.pl).

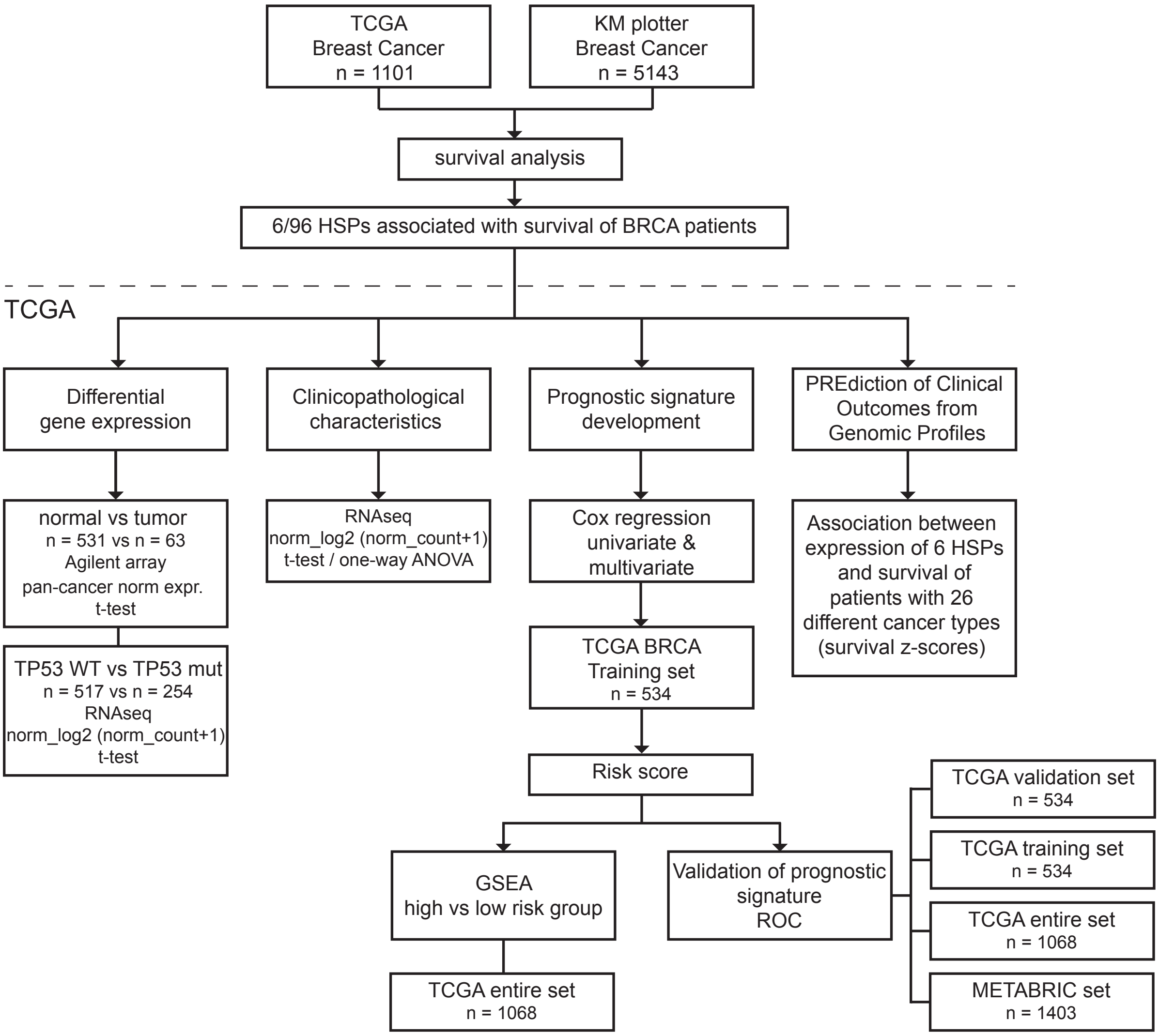


**Supplementary Fig. S1.** Workflow of the present study.

| Gene | Coefficient | Coeficient SE | P-value |
|---|---|---|---|
| **CCT1** | **0,52** | **0,13** | **0,0001** |
| **DNAJC23** | **0,42** | **0,12** | **0,0005** |
| **HSPA9** | **0,54** | **0,16** | **0,0007** |
| **HSP90AA1** | **0,38** | **0,11** | **0,0009** |
| **HSPA8** | **0,41** | **0,13** | **0,0012** |
| **DNAJB5** | **-0,30** | **0,11** | **0,0064** |
| **HSPA2** | **-0,13** | **0,05** | **0,0065** |
| **HSPH1** | **0,26** | **0,10** | **0,0130** |
| **CCT4** | **0,39** | **0,16** | **0,0140** |
| **DNAJC13** | **0,39** | **0,16** | **0,0160** |
| **HSCB** | **-0,29** | **0,13** | **0,0330** |
| **DNAJC30** | **-0,31** | **0,15** | **0,0360** |
| **CCT5** | **0,25** | **0,12** | **0,0440** |
| **CCT2** | **0,24** | **0,12** | **0,0460** |
| **DNAJC4** | **-0,22** | **0,11** | **0,0500** |
| CCT8 | 0,29 | 0,16 | 0,0630 |
| HSPD1 | 0,21 | 0,12 | 0,0720 |
| HSP90AB1 | 0,24 | 0,13 | 0,0740 |
| HSPB8 | 0,07 | 0,04 | 0,0760 |
| HSPA4 | 0,28 | 0,16 | 0,0770 |
| DNAJA2 | 0,24 | 0,14 | 0,0850 |
| DNAJB13 | -0,12 | 0,08 | 0,1100 |
| CCT7 | 0,28 | 0,18 | 0,1200 |
| DNAJB12 | -0,30 | 0,19 | 0,1300 |
| DNAJC6 | 0,07 | 0,05 | 0,1500 |
| HSPA14 | 0,16 | 0,13 | 0,2000 |
| DNAJB8 | 0,56 | 0,44 | 0,2000 |
| DNAJC5 | 0,19 | 0,15 | 0,2000 |
| HSPA7 | -0,07 | 0,06 | 0,2100 |
| HSPA13 | 0,13 | 0,10 | 0,2100 |
| HSPE1 | 0,15 | 0,12 | 0,2100 |
| DNAJB14 | 0,14 | 0,11 | 0,2100 |
| DNAJC14 | 0,26 | 0,21 | 0,2100 |
| DNAJC12 | -0,04 | 0,03 | 0,2400 |
| HSPB4 | 0,14 | 0,12 | 0,2500 |
| HSPB11 | -0,15 | 0,13 | 0,2500 |
| DNAJB7 | -0,12 | 0,11 | 0,2500 |
| DNAJC1 | -0,09 | 0,08 | 0,2500 |
| DNAJC22 | 0,05 | 0,04 | 0,2500 |
| DNAJA4 | 0,09 | 0,08 | 0,2600 |
| HSPA6 | -0,07 | 0,07 | 0,2700 |
| DNAJC11 | 0,22 | 0,21 | 0,2800 |
| DNAJC21 | 0,20 | 0,19 | 0,2800 |
| CCT3 | 0,15 | 0,14 | 0,3000 |
| DNAJC28 | -0,12 | 0,11 | 0,3100 |
| DNAJC3 | 0,13 | 0,13 | 0,3200 |
| DNAJC19 | -0,14 | 0,14 | 0,3200 |
| DNAJC17 | -0,11 | 0,12 | 0,3300 |

| Gene | Coefficient | Coeficient SE | P-value |
|---|---|---|---|
| HSPB3 | 0,07 | 0,07 | 0,3600 |
| HSPB5 | -0,04 | 0,04 | 0,3600 |
| DNAJC15 | -0,09 | 0,10 | 0,3600 |
| DNAJC2 | -0,14 | 0,15 | 0,3700 |
| DNAJC16 | -0,13 | 0,15 | 0,3800 |
| CCT6A | 0,11 | 0,13 | 0,3900 |
| DNAJC27 | 0,13 | 0,16 | 0,4000 |
| HSPB2 | -0,05 | 0,06 | 0,4100 |
| DNAJB4 | 0,08 | 0,10 | 0,4200 |
| HYOU1 | 0,09 | 0,12 | 0,4400 |
| HSPA12B | -0,05 | 0,07 | 0,4500 |
| DNAJB3 | 0,15 | 0,22 | 0,5000 |
| DNAJA3 | 0,11 | 0,17 | 0,5100 |
| HSPA4L | 0,03 | 0,05 | 0,5200 |
| DNAJC7 | -0,11 | 0,18 | 0,5300 |
| DNAJC24 | -0,11 | 0,17 | 0,5300 |
| HSPB6 | -0,03 | 0,05 | 0,5500 |
| DNAJC25 | 0,11 | 0,18 | 0,5500 |
| DNAJB2 | 0,08 | 0,13 | 0,5600 |
| HSPB10 | -0,14 | 0,27 | 0,5900 |
| CCT6B | -0,05 | 0,09 | 0,6000 |
| MKKS | 0,10 | 0,19 | 0,6000 |
| HSPB7 | 0,02 | 0,05 | 0,6200 |
| DNAJC5B | -0,03 | 0,05 | 0,6200 |
| DNAJC29 | -0,04 | 0,08 | 0,6200 |
| DNAJC8 | -0,11 | 0,23 | 0,6300 |
| DNAJC26 | 0,07 | 0,15 | 0,6300 |
| BBS12 | -0,06 | 0,12 | 0,6500 |
| DNAJA1 | 0,06 | 0,15 | 0,6700 |
| DNAJB9 | 0,05 | 0,13 | 0,6800 |
| BBS10 | -0,05 | 0,13 | 0,7100 |
| HSPA12A | 0,03 | 0,08 | 0,7400 |
| DNAJB11 | -0,05 | 0,14 | 0,7400 |
| HSPA1B | 0,03 | 0,10 | 0,7800 |
| DNAJC10 | 0,04 | 0,14 | 0,7900 |
| DNAJB6 | -0,03 | 0,16 | 0,8300 |
| HSPB1 | 0,01 | 0,06 | 0,8400 |
| HSP90L | 0,02 | 0,12 | 0,8600 |
| HSPA1A | 0,01 | 0,09 | 0,8900 |
| DNAJC18 | -0,02 | 0,13 | 0,8900 |
| HSP90B1 | -0,02 | 0,16 | 0,9000 |
| DNAJB1 | 0,02 | 0,15 | 0,9100 |
| HSPA1L | -0,01 | 0,13 | 0,9200 |
| HSPB9 | 0,01 | 0,08 | 0,9200 |
| DNAJC5G | 0,03 | 0,29 | 0,9300 |
| HSPA5 | 0,01 | 0,15 | 0,9600 |
| DNAJC9 | 0,00 | 0,15 | 0,9800 |

**Supplementary Fig. S2.** Univariate Cox proportional hazards analysis of overall survival for TCGA BRCA patients based on HSP expression.

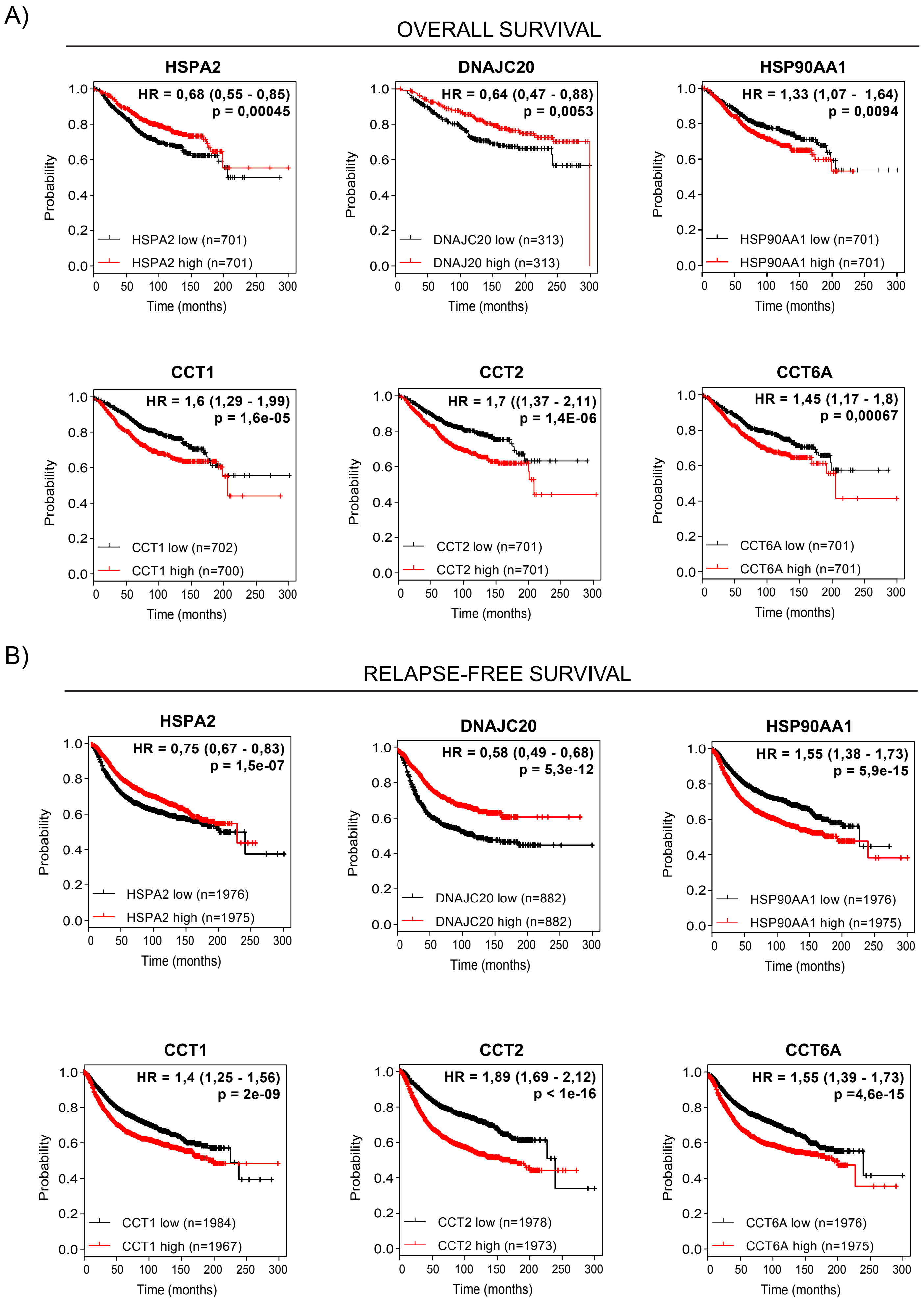


**Supplementary Fig. S3.** Expression of six identified HSPs predicts survival of breast cancer patients. Kaplan-Meier analyses of overall survival **(A)** and relapse-free survival **(B)** based on the gene expression in cohort of KM plotter patients. Hazard ratios (HR) with 95% confidence intervals and p-values (log-rank test, Mantel-Haenszel) were calculated. Data for survival curves were obtained from KM plotter (http://kmplot.com/analysis/).

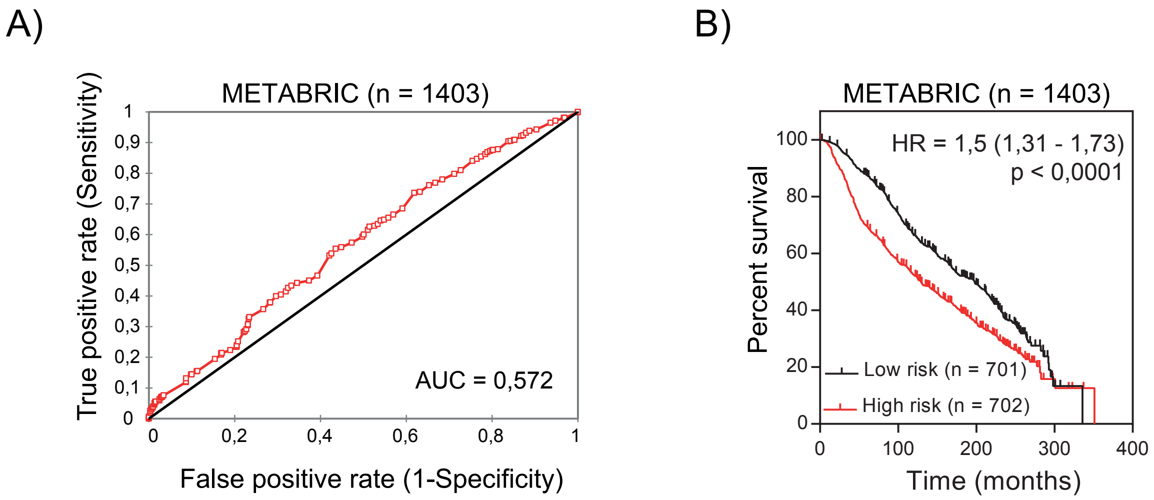


**Supplementary Fig. S4. (A)** Diagnostic value of five candidate HSPs and cancer stage in METABRIC dataset (n = 1403). The areas under curve (AUC) were calculated for ROC curves, and sensitivity and specificity were calculated to assess the score performance. **(B)** Kaplan-Meier survival curves for five-HSP and stage signature in METABRIC dataset (n = 1403). Patients were stratified into high-risk and low-risk groups based on median of risk score. Hazard ratios (HR) with 95% confidence intervals and log-rank p-values were calculated.

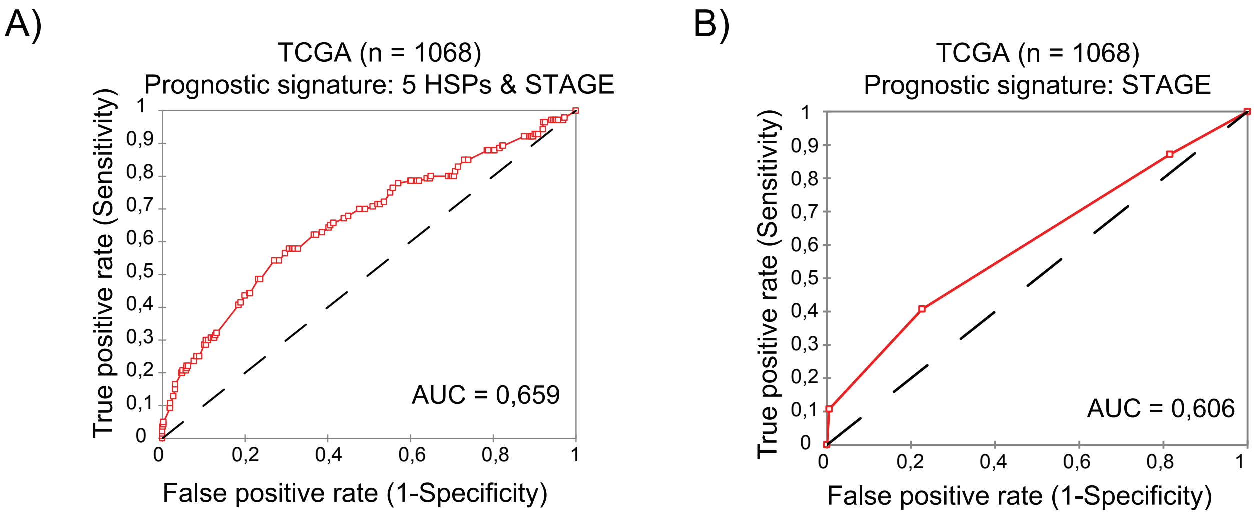


**Supplementary Fig. S5.** Comparison of signatures for survival prediction of TCGA breast cancer patients based on **A)** expression of 5 HSPs and stage, **B)** stage only. The areas under curve (AUC) were calculated for ROC curves, and sensitivity and specificity were calculated to assess the score performance.

A)

| Multivariate analysis | | | | | |
|---|---|---|---|---|---|
| **Variable** | **Coefficient** | **p-value** | **HR** | **95% CI** | |
| | | | | **Lower** | **Upper** |
| **Training set (n = 534)** | | | | | |
| HSPA2 | -0,461 | 0,084 | 0,631 | 0,374 | 1,063 |
| DNAJC20 | -0,180 | 0,502 | 0,835 | 0,494 | 1,413 |
| HSP90AA1 | 0,703 | **0,036** | 2,020 | 1,048 | 3,896 |
| CCT1 | 0,127 | 0,661 | 1,135 | 0,644 | 2,002 |
| CCT2 | 0,160 | 0,625 | 1,173 | 0,619 | 2,223 |
| STAGE I | -3,357 | **< 0,0001** | 0,035 | 0,011 | 0,111 |
| STAGE II | -3,238 | **< 0,0001** | 0,039 | 0,014 | 0,107 |
| STAGE III | -2,426 | **< 0,0001** | 0,088 | 0,032 | 0,249 |
| **Validation set (n = 534)** | | | | | |
| HSPA2 | -0,625 | **0,015** | 0,535 | 0,323 | 0,887 |
| DNAJC20 | -0,064 | 0,791 | 0,938 | 0,583 | 1,509 |
| HSP90AA1 | 0,391 | 0,146 | 1,478 | 0,873 | 2,502 |
| CCT1 | 0,558 | **0,0315** | 1,747 | 1,051 | 2,906 |
| CCT2 | -0,1416 | 0,584 | 0,868 | 0,523 | 1,441 |
| STAGE I | -2,7548 | **< 0,0001** | 0,064 | 0,025 | 0,165 |
| STAGE II | -2,3064 | **< 0,0001** | 0,010 | 0,046 | 0,218 |
| STAGE III | -1,7115 | **< 0,0001** | 0,180 | 0,082 | 0,397 |
| **Entire TCGA set (n = 1068)** | | | | | |
| HSPA2 | -0,549 | **0,0025** | 0,578 | 0,405 | 0,824 |
| DNAJC20 | -0,123 | 0,492 | 0,885 | 0,624 | 1,255 |
| HSP90AA1 | 0,509 | **0,014** | 1,663 | 1,080 | 2,495 |
| CCT1 | 0,401 | **0,035** | 1,494 | 1,029 | 2,170 |
| CCT2 | -0,0005 | 0,998 | 1,001 | 0,677 | 1,478 |
| STAGE I | -2,807 | **< 0,0001** | 0,060 | 0,030 | 0,123 |
| STAGE II | -2,548 | **< 0,0001** | 0,078 | 0,043 | 0,143 |
| STAGE III | -1,891 | **< 0,0001** | 0,150 | 0,082 | 0,279 |

HR, hazard ratio; CI, confidence interval

B)

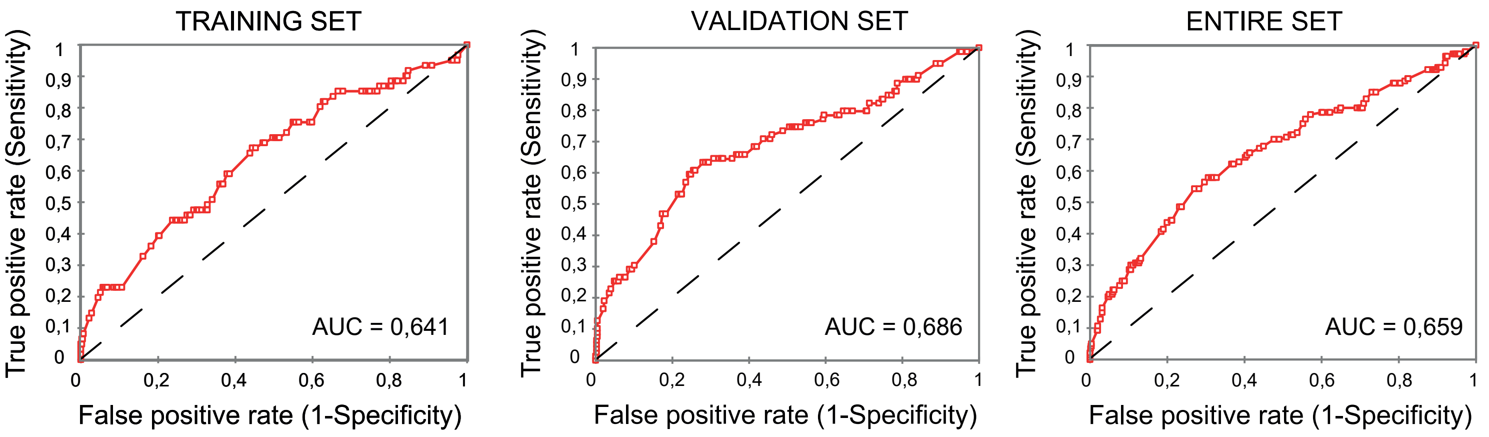


C)

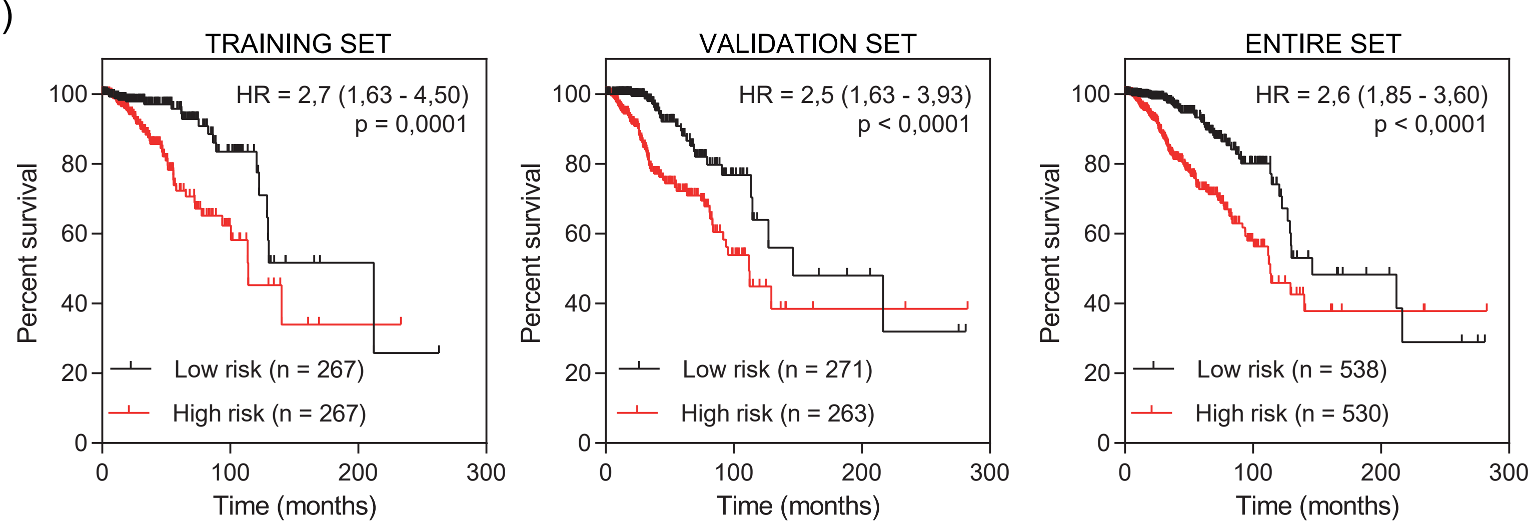


**Supplementary Fig. S6.** Survival prediction model for breast cancer patients calculated using formula with binarized Stage variables:
Risk score = (-0,461 x HSPA2 0/1) + (-0,180 x DNAJC20 0/1) + (0,703 x HSP90AA1 0/1) + (0,127 x CCT1 0/1) + (0,16 x CCT2 0/1) + (-3,357 x Stage I 0/1) + (-3,238 x Stage II 0/1) + (-2,426 x Stage III 0/1).
Expression data were converted to a binary format as follows: low expression = 0, high expression = 1 and stage was binarized as follows: e.g. Stage I NO = 0, YES = 1.
**(A)** Multivariate Cox proportional hazards analysis of overall survival for TCGA BRCA patients. **(B)** Diagnostic value of five candidate HSPs and cancer stage (I/II/III) in the training (n = 534), validation (n = 534) and entire TCGA BRCA dataset (n = 1068). The areas under curve (AUC) were calculated for ROC curves, and sensitivity and specificity were calculated to assess the score performance. **(C)** Kaplan-Meier survival curves for five-HSP and stage signature in the training (n = 534), validation (n = 534) and entire TCGA BRCA dataset (n = 1068). Patients were stratified into high-risk and low-risk groups based on median of risk score. Hazard ratios (HR) with 95% confidence intervals and log-rank test p-values were calculated.
